# TGF-β1-Induced Upregulation of MALAT1 Promotes Kazakh's Esophageal Squamous Cell Carcinoma Invasion by EMT

**DOI:** 10.7150/jca.48426

**Published:** 2020-10-04

**Authors:** Qing Liu, Shutao Zheng, Yumei Chen, Tao Liu, Xiujuan Han, Xiao Zhang, Tongxue Shen, Xiaomei Lu

**Affiliations:** 1 Clinical Medical Research Institute, First Affiliated Hospital of Xinjiang Medical University, Xinjiang Uygur Autonomous Region, Urumqi, PR China.; 2 State Key Laboratory of Pathogenesis, Prevention, Treatment of High Incidence Diseases in Central Asian, Xinjiang Uygur Autonomous Region, Urumqi, PR China.; 3 Health Management Center, Xinjiang Medical University, Xinjiang Uygur Autonomous Region, Urumqi, PR China.

**Keywords:** Kazakh's ESCC, EMT, TGF-β1, MALAT1

## Abstract

Transforming growth factor β1 (TGF-β1) plays an important role in tumor initiation and development by inducing epithelial-mesenchymal Transition (EMT). Metastasis-Associated Lung Adenocarcinoma Transcript 1 (MALAT1) is a long noncoding RNA (lncRNA) that contributes to the invasion and metastasis of tumors, including esophageal squamous cell carcinoma (ESCC). The aim of the present study was to explore the underlying mechanisms implicated in EMT and to clarify whether TGF-β1 regulates MALAT1 expression, thereby promoting the invasion of ESCC. Expression of TGF-β1, MALAT1 and EMT-related markers, including E-cadherin and Vimentin, was detected in clinical samples of Kazakh's ESCC. The role of TGF-β1 in the regulation of MALAT1 in ESCC invasion was evaluated at the ESCC cell line level. High TGF-β1 expression was significantly associated with poor survival among patients with Kazakh's ESCC. Additionally, the expression of Vimentin was upregulated, and the expression of E-cadherin was downregulated and varied. The expression of MALAT1 positively correlated with the expression of TGF-β1 both *in vivo* and *in vitro*. Furthermore, knockdown of MALAT1 inhibited TGF-β1-induced EMT. Our data indicate that MALAT1 is heavily involved in EMT induced by TGF-β1. MALAT1 may be a therapeutic target in the suppression of metastasis and invasion of ESCC.

## Introduction

Esophageal cancer (EC) is the eighth most aggressive cancer and is the sixth leading cause of cancer death globally 1, 2. The dominant type of EC worldwide is esophageal squamous cell carcinoma (ESCC), with the highest incidence rates in populations within Southeastern and Central Asia 3, 4. People of the Kazakh ethnicity in Xinjiang, China, have the highest incidence of ESCC 5. Unless diagnosed at a very early stage, patients present with widespread metastasis where current treatment is largely ineffective 6, 7. Therefore, understanding the specific causes of metastasis in patients with ESCC is essential for improving the prognosis of ESCC.

Epithelial-to-mesenchymal transition (EMT) plays a critical role in driving cancer cell metastasis 8, 9. Transforming growth factor β (TGF-β1) is the driver of EMT, and many influencing factors promote EMT through this signaling pathway 10, 11. Recently, several reports revealed that lncRNA Metastasis-Associated Lung Adenocarcinoma Transcript 1 (MALAT1) has crucial functions in malignant cancer progression 12. Extensive evidence has shown that MALAT1 is aberrantly expressed in a broad range of human malignancies, including lung cancer 13, 14, pancreatic cancer 15, 16, cervical cancer 17, breast cancer 18, 19, osteosarcoma 20 and ESCC 12. MALAT1 regulates tumor progression by various signaling pathways and mechanisms, particularly EMT 21, 22. Although various functions have been described for MALAT1 in many different cancers, the mechanism by which MALAT1 regulates EMT in ESCC remains unclear.

Based on recent studies, we postulated that MALAT1 may promote the invasion of ESCC through TGF-β1-induced EMT. Therefore, the expression levels of TGF-β1, MALAT1 and EMT markers in ESCC specimens were evaluated, and their correlations were validated. The role of MALAT1 in regulating ESCC metastasis at the cellular level was also evaluated. These results indicate that MALAT1 is an important mediator of TGF-β1-induced EMT in ESCC.

## Materials and Methods

### Patients and tissues

Forty-three pairs of fresh frozen samples, as well as seventy-two pairs of formalin-fixed paraffin-embedded (FFPE) timorous and healthy adjacent tissues of Kazakh's ESCC, were obtained from the First Affiliated Hospital of Xinjiang Medical University. Primary ESCC tissue areas and normal adjacent tissue (NAT) from the same patients were separately excised by experienced pathologists. The study was approved by the local research ethical committee, and signed informed consent was obtained from all the patients. None of the patients received any treatment before surgery.

### qRT-PCR

qRT-PCR was performed to detect the expression of TGF-β1 and MALAT1 in tissue specimens. After normalization with GAPDH, relative gene expression was calculated with the 2^-ΔΔct^ method. The primer sequences used in this study were as follows: TGF-β1, F: 5'-GCGACTCGCCAGAGTGGTTA-3', R: 5'-GTTGATGTCCACTTGCAGTGTGTTA-3'; E-cadherin, F: 5'-TCGCTTACACCATCCTCAGC-3', R: 5'-AGGGAAACTCTCTCGGTCCA-3'; Vimentin, F: 5'-GAAGAGAACTTTGCCGTTGAAG-3', R: 5'-GAAGGTGACGAGCCATTTCC-3'; MALAT1, F: 5'-GCAGACCCAGAGCAGTGTAA-3', R: 5'-AAACGCCTCAATCCCACA-3'; and GAPDH, F: 5'-GACTCATGACCACAGTCCATGC-3', R: 5'-AGAGGCAGGGATGATGTTCTG-3'. All reactions were performed independently in triplicate.

### Immunohistochemistry (IHC)

The expression of TGF-β1, E-cadherin and Vimentin in 72 pairs of ESCC samples was detected by IHC. After deparaffinization and antigen retrieval, sections were incubated at 4 °C overnight with primary antibodies at a specified dilution (TGF-β1 1:50, Santa Cruz, E-cadherin 1:50, Cell Signaling Technology, Vimentin 1:100, Cell Signaling Technology, USA). Then, sections were incubated with secondary antibody at a 1:800 dilution (Abcam, USA) for 60 min at 37 °C. Finally, sections were incubated with streptavidin-horseradish peroxidase complex and stained with diaminobenzidine Kit (Beijing Zhongshan Golden Bridge Biotechnology Co, Ltd). Staining was assessed with regard to intensity (0: no intensity; 1: weak intensity; 2: moderate intensity; 3: strong intensity) and percentage (0: less than 10%; 1: between 10% - 25%; 2: between 25% - 50%; 3: more than 50%) of positive cells. Based on the total score (staining intensity plus positive cell score), the results were classified as the 'negative expression' group (total score: 0-2) and the 'positive expression' group (total score: 3-6).

### Western blot

Western blotting was performed to detect the expression of TGF-β1, E-cadherin and Vimentin as previously described 7. The primary antibody dilutions were 1:500 for TGF-β1, 1:800 for E-cadherin and Vimentin, and 1:1000 for GAPDH. Protein expression was determined using Odyssey infrared dual color imaging.

### RNA interference

The ESCC cell lines Eca109 and Ec9706 were purchased from Wu Han University (Wu Han, China), and KYSE30, KYSE150, KYSE450 and KYSE510 were kind gifts from Professor Zhihua Liu. All the cell lines were cultured in 1640 medium with 10% FBS and incubated at 37°C with 5% CO_2_.

The following three siRNAs for MALAT1 were transfected into cells at a concentration of 20 nM with Lipofectamine 3000 (Invitrogen, USA): MALAT1 siRNA-1, sense: 5'-GATCCATAATCGGTTTCAAGG-3', antisense: 5'-TTGAAACCGATTATGGATCAT-3'; MALAT1 siRNA-2, sense: 5'-CACAGGGAAAGCGAGTGGTTGGTAA-3', antisense: 5'-TTACCAACCACTCGCTTTCCCTGTG-3'; MALAT1 siRNA-3, sense: 5'-CAGACAGGTATCTCTTCGTTA-3', antisense: 5'-GTCTGTCCATAGAGAAGCAAT-3'; and MALAT1 siRNA-scramble sense: 5'-UUCUCCGAACGUGUCACGUTT-3', antisense: 5'-ACGUGACACGUUCGGAGAATT-3' (GenePharma, Shanghai, China). The negative control was transfected with scramble mimics. Cells were incubated with siRNAfor6 h at 37 °C and then cultured in complete medium until the designated time points.

### Immunofluorescence (IF)

Cells were plated onto glass bottom cell culture dishes (NEST, 801001). After treatment with recombinant TGF-β1 or TGF-β1 inhibitor, cells were washed with PBS and fixed with 95% ethanol. Then, 0.5% Triton X-100 was added before incubation with anti-TGF-β1 or E-cadherin antibodies (1:200, Abcam Corporation). After washing with PBS, the cells were incubated with anti-rabbit IgG Fab2 Alexa Fluor 494 (rabbit)-conjugated secondary antibody (Cell Signaling Technology) for 2 h at room temperature. The nuclei were simultaneously counterstained with Hoechst 33258 (0.05 mg/mL, Santa Cruz Biochemical). Expression of indicated proteins in cells was observed and quantified by fluorescent confocal microscopy (LeicaTCSSP8).

### Cell proliferation assay

Cells were plated at a density of 4×10^3^ cells per well in 96-well plates. MTT assays were performed at indicated time points. The absorbance was recorded at 490 nm. Relative cellular growth was determined based the ratio of the average absorbance in treated cells to that in control cells.

### Wound healing assay

Cells were plated into 6-well plates and cultured for 24 h. After transfection with siRNA, a perpendicular linear scratch was made using a sterile pipette tip. The wound was measured at the indicated times.

### Transwell assays

Transwell assays of cell invasion were performed as previously described 23. Cells found on the lower surfaces of the Matrigel-coated membranes indicated invasion, and the number of cells was counted under a light microscope.

### Apoptosis

The Annexin V staining procedure was followed according to the instructions provided by the manufacturer (Invitrogen, USA). Cells were analyzed by flow cytometry (FCM) in triplicate.

### Statistical analysis

Statistical analysis was performed using SPSS version 17.0 software. Differences between groups were calculated with Student's *t*-test. Associations between protein expression and pathological parameters were assessed by the Pearson chi-square test. Overall survival curves were plotted according to the Kaplan-Meier survival function with a log-rank test. A *P* value less than 0.05 was considered statistically significant.

## Results

### TGF-β1 induced EMT, causing invasion in ESCC

**Figure 1:**
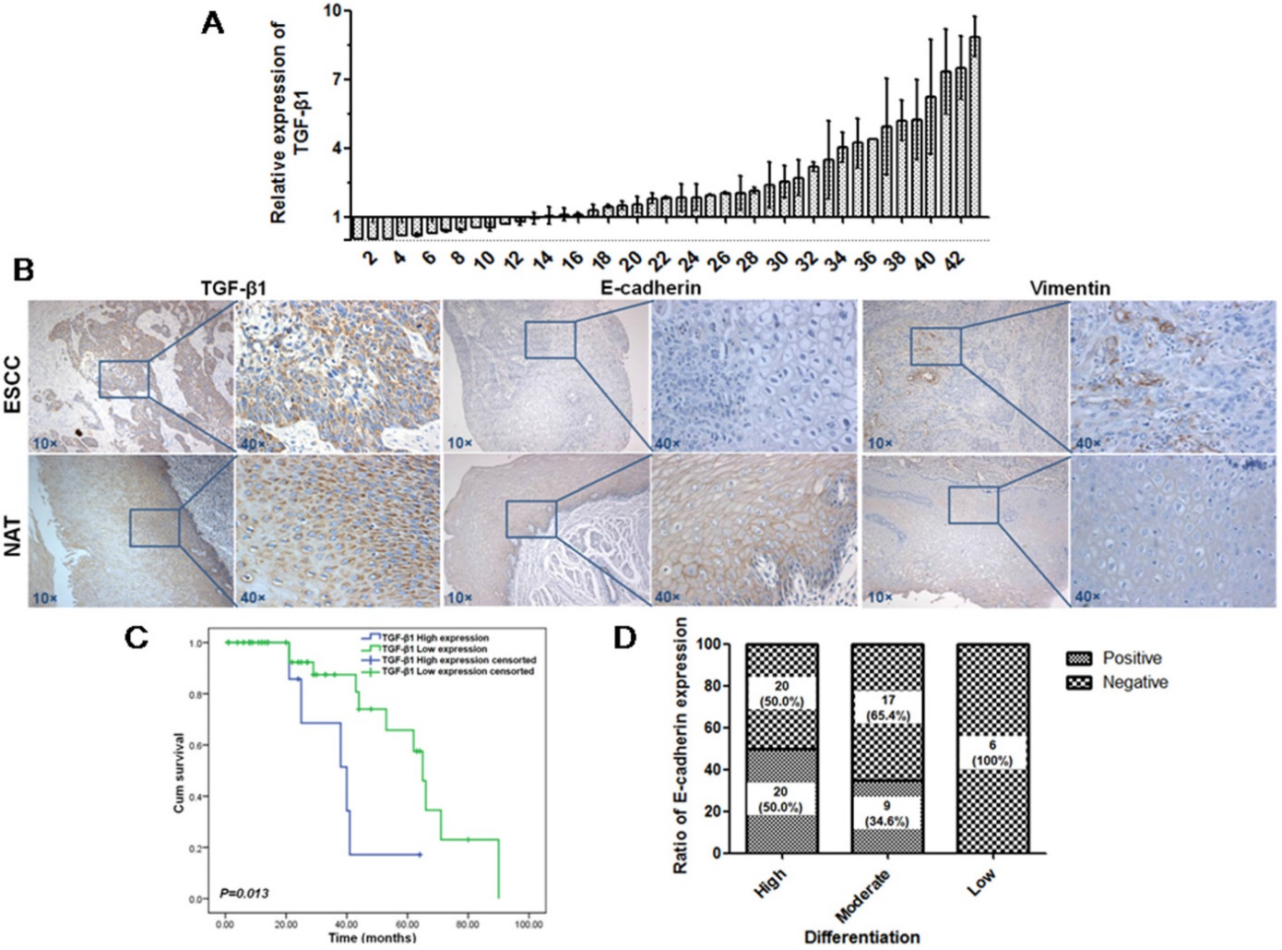
** Expression of TGF-β1, Vimentin and E-cadherin in patients with ESCC.** (A) Expression of TGF-β1 in ESCC tissues was detected by qRT-PCR. (B) Expression of TGF-β1, Vimentin and E-cadherin in another cohort was detected by IHC. Magnification: 100×, 400×. (C) Kaplan-Meier overall survival curves for 72 patients with ESCC stratified by high and low expression of TGF-β1. (D) Expression of E-cadherin was significantly associated with tumor differentiation.

**Figure 2:**
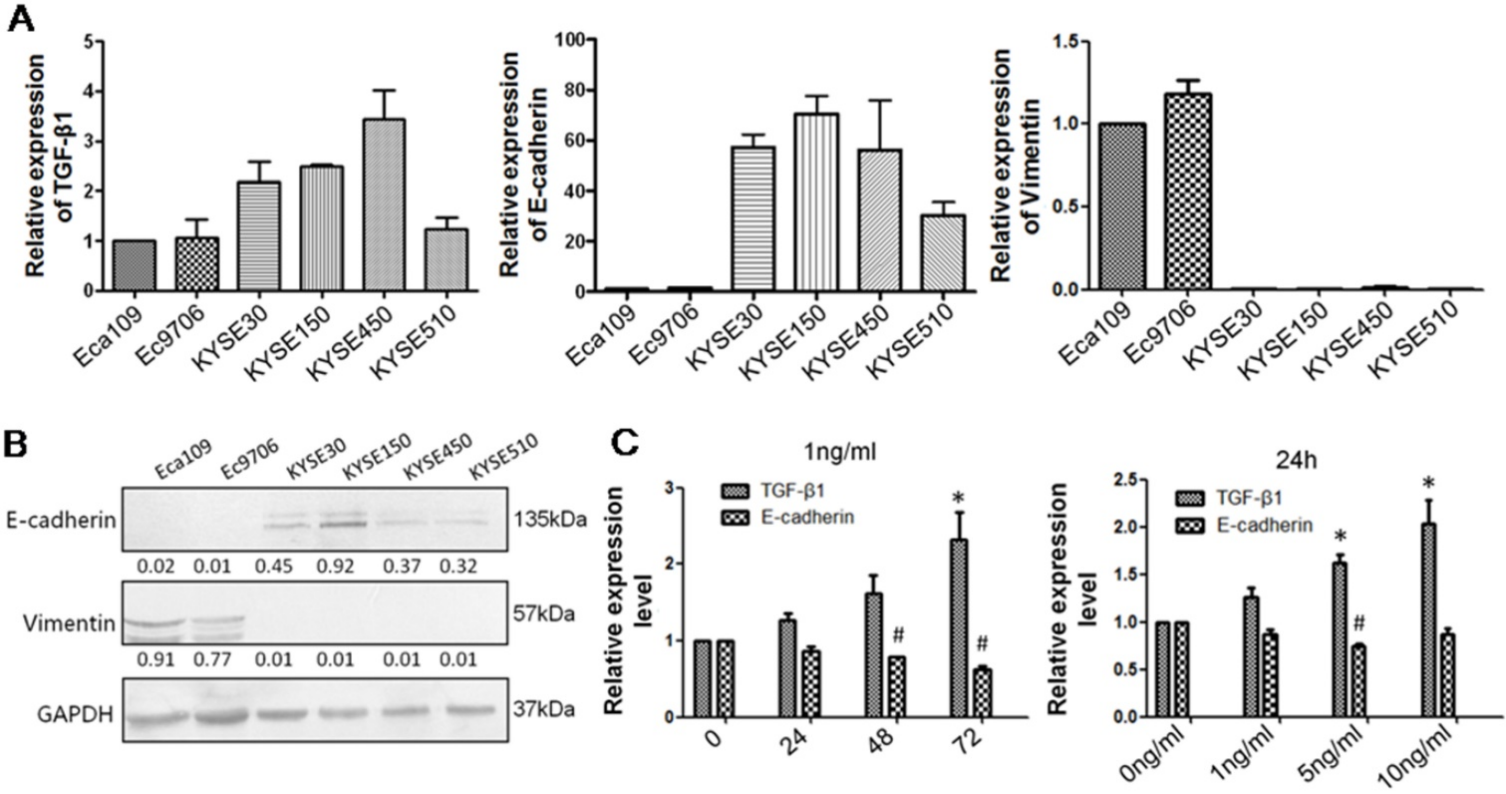
** TGF-β1 negatively regulates E-cadherin expression.** (A) qRT-PCR was applied to measure the levels of TGF-β1, Vimentin and E-cadherin in six ESCC cell lines: Eca109, Ec9706, KYSE30, KYSE150, KYSE450, and KYSE510. (B) Western blot analysis showed that the KYSE150 cell line expressed high levels of E-cadherin and that Eca109 expressed high levels of Vimentin. (C) Recombinant TGF-β1 regulated E-cadherin expression over time and with increasing concentration. *: Compared with the control group, TGF-β1 was significantly upregulated (*P<0.05*). #: Compared with the control group, E-cadherin was significantly reduced (*P*<0.05).

**Figure 3:**
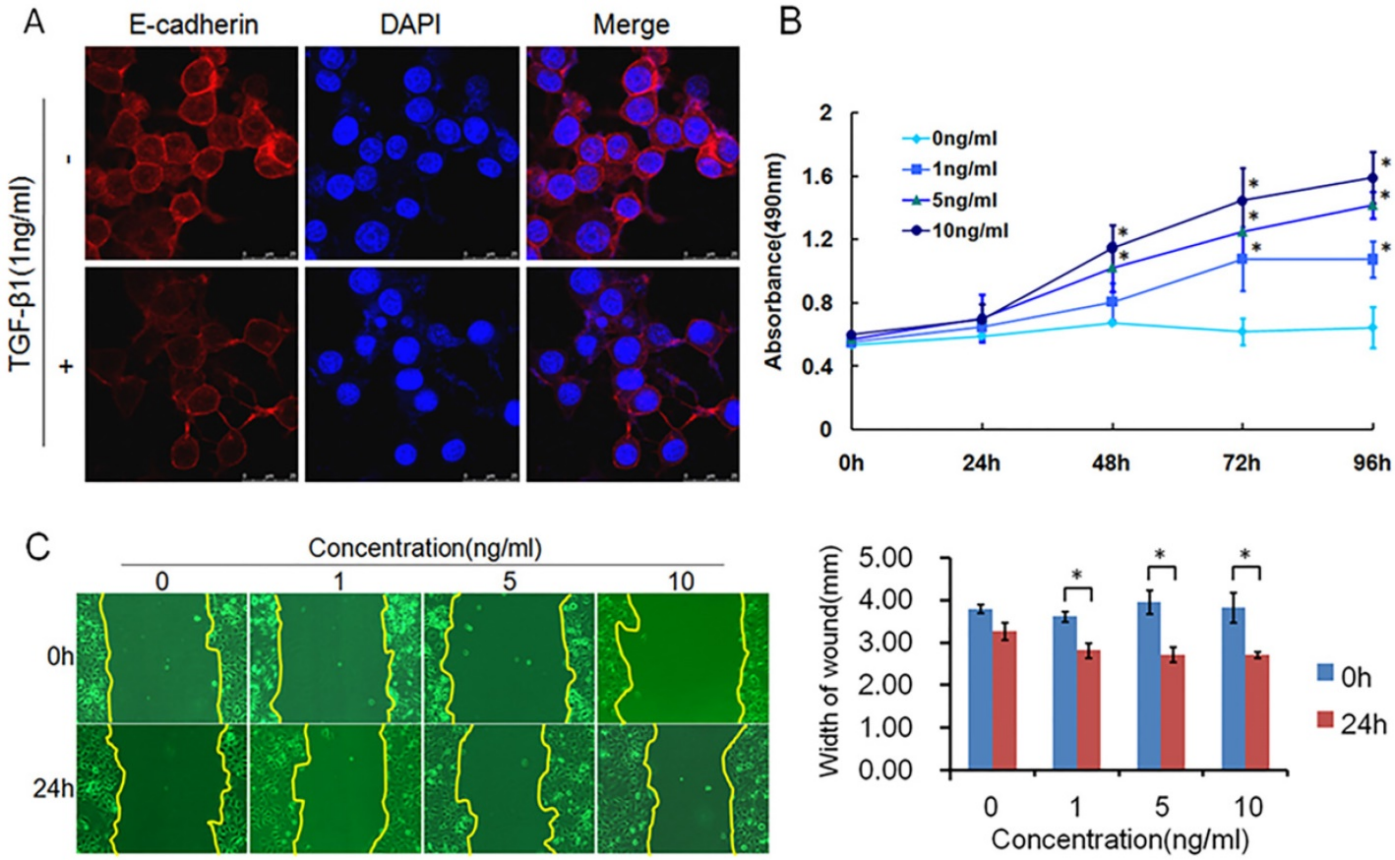
** TGF-β1 induced EMT, causing migration in ESCC.** (A) Immunofluorescence showed that the expression of E-cadherin on the cell membrane was obviously reduced after treatment with recombinant TGF-β1. (B) MTT assays revealed that recombinant TGF-β1 significantly increased the growth rate of ESCC cells. (C) Migratory variation of ESCC cells was analyzed using a wound healing assay after treatment with recombinant TGF-β1. Magnification: 100× (*, P<0.05).

**Figure 4:**
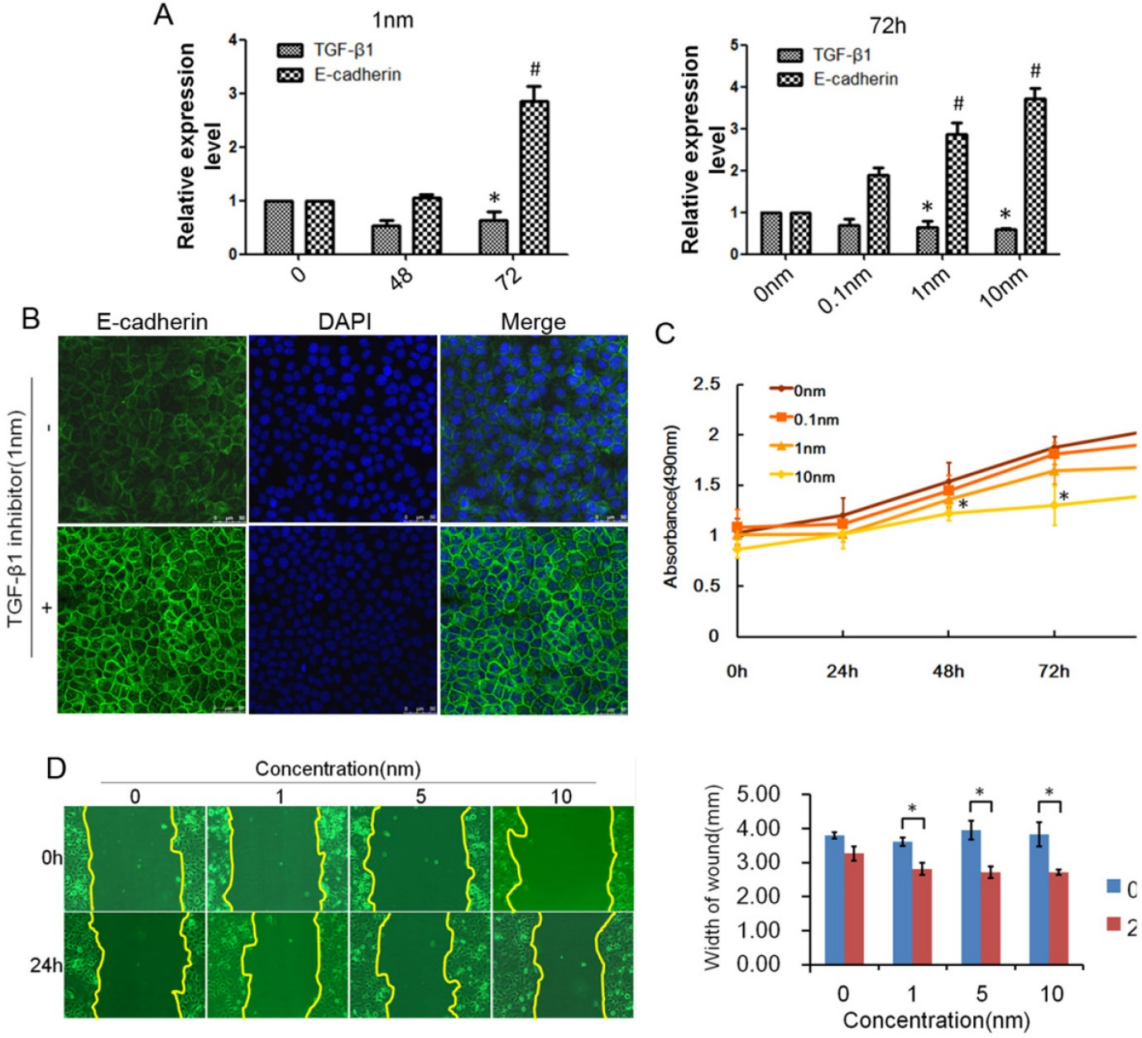
** Inhibited expression of TGF-β1 reduced EMT in ESCC.** (A) E-cadherin expression could be regulated by TGF-β1 inhibitor over time and with increasing concentration. (B) Immunofluorescence showed that the expression of E-cadherin on the cell membrane was obviously increased after treatment with TGF-β1 inhibitor. (C) MTT assays revealed that TGF-β1 inhibitor significantly decreased the growth rate of ESCC cells. (D) The migratory variations of ESCC cells were analyzed using a wound healing assay after treatment with TGF-β1 inhibitor. Magnification: 100× (*, P<0.05).

TGF-β1 plays a dual role in cancer progression. It suppresses tumor growth at the early stage but, promotes tumor invasion and metastasis at the progressive stage 24. Recent studies have shown that TGF-β1 induces tumor progression through EMT 25, 26. Therefore, we first confirmed the expression of TGF-β1 and EMT markers in ESCC specimens. Upregulated expression of TGF-β1 was found in 30 of 43 ESCC tissues compared with adjacent normal tissues (Fig. 1A). In a different cohort of Kazakh ethnicity, significantly high levels of TGF-β1 and Vimentin expression were found in ESCC tissues compared with adjacent normal tissues, while the expression of E-cadherin was significantly low in ESCC tissues (Fig. 1B, Table 1). Furthermore, there was a correlation between TGF-β1 and patient survival (Fig. 1C), and patients with high levels of TGF-β1 expression had a poor prognosis. The expression of E-cadherin was significantly associated with tumor differentiation (Fig. 1D, Table 2), and the frequency of E-cadherin expression was reduced when differentiation became poor. We next examined E-cadherin expression levels and cell function changes in ESCC cells after treatment with recombinant TGF-β1 or TGF-β1 inhibitor. Higher expression of TGF-β1 significantly correlated with decreased expression of E-cadherin over time and with increasing concentration (Fig. 2C). Fig. 3A shows that the expression of E-cadherin on the cell membrane was obviously reduced after treatment with TGF-β1. Additionally, cell proliferation was increased, and cell migration was enhanced upon TGF-β1 treatment (Fig. 3B&C). On the other hand, the expression of E-cadherin was obviously increased after ESCC cells were treated with a TGF-β1 inhibitor (Fig. 4A&B). Furthermore, cell proliferation was decreased, and cell migration was weakened when TGF-β1 expression was inhibited (Fig. 4C&D). These results indicate that EMT occurs in ESCC and that TGF-β1 may be a vital influencing factor of EMT.

### MALAT1 was positively associated with TGF-β1

**Figure 5:**
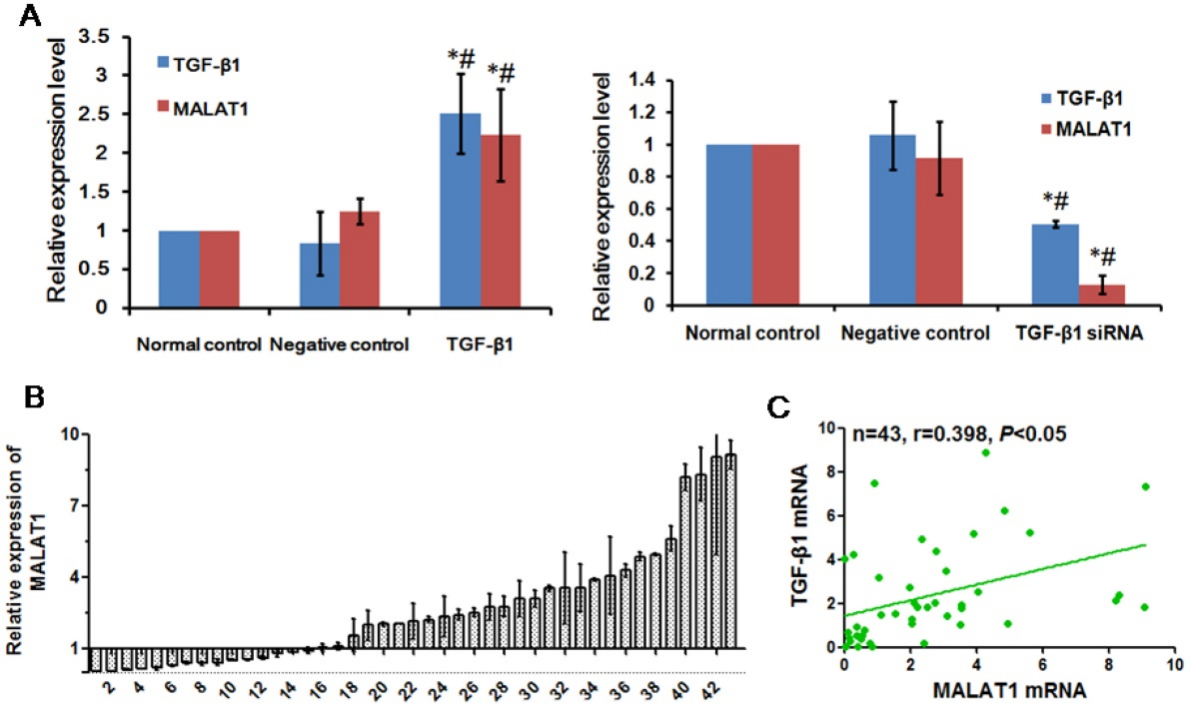
** MALAT1 was positively associated with TGF-β1.** (A) Expression of MALAT1 after TGF-β1 or TGF-β1 siRNA transfection was detected by qRT-PCR. (B) Expression of MALAT1 in ESCC tissues was detected by qRT-PCR. (C) Correlation between expression of MALAT1 and TGF-β1 in ESCC tissues. *: Compared with that in the normal control group, the expression of TGF-β1 and MALAT1 in the TGF-β1 siRNA group was significantly changed (*P*<0.05). #: Compared with the negative control group, the expression of TGF-β1 and MALAT1 in the TGF-β1 siRNA group was significantly changed (*P<*0.05).

MALAT1 is a lncRNA associated with EMT in various cancers 27. To determine whether MALAT1 was associated with EMT induced by TGF-β1, we examined the expression of MALAT1 after TGF-β1 treatment. There was a positive correlation between TGF-β1 and MALAT1, as shown in Fig. 5A. When TGF-β1 was transfected into ESCC cells overexpressing TGF-β1, the expression of MALAT1 was also significantly upregulated. Additionally, the expression of MALAT1 was significantly reduced after transfection with TGF-β1 siRNA. To confirm the correlation between TGF-β1 and MALAT1, we examined the expression of MALAT1 in ESCC specimens. A high level of MALAT1 expression was found in 28 of 43 ESCC tissues compared with adjacent normal tissues (Fig. 5B). Furthermore, there was a positive correlation between the expression of MALAT1 and TGF-β1 in ESCC tissues (Fig. 5C, r=0.398, *P*<0.05).

### MALAT1 promoted the invasion of ESCC via TGF-β1-induced EMT

**Figure 6:**
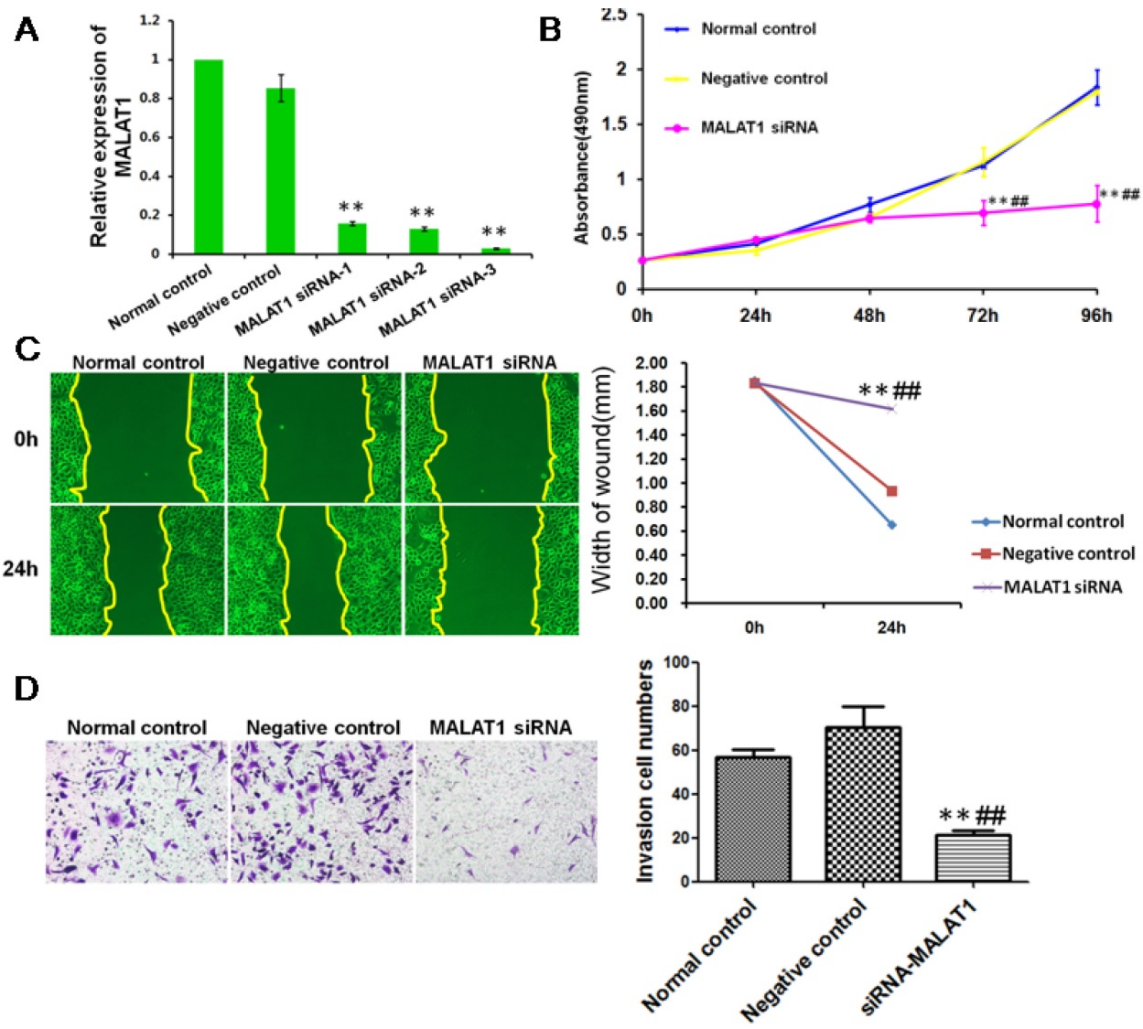
** MALAT1 promoted invasion of ESCC.** (A) qRT-PCR showed that MALAT1 siRNA significantly reduced the expression of MALAT1 in ESCC cells. (B) MTT assays revealed that MALAT1 knockdown significantly reduced the growth rate of ESCC cells. (C) Migratory variation of ESCC cells was analyzed using a wound healing assay after MALAT1 knockdown. Magnification: 100×. (D) Transwell assay showing the variations in ESCC cell invasion after MALAT1 knockdown. (**: compared with normal control,* P*<0.01; ##: compared with negative control, *P<*0.01).

**Figure 7:**
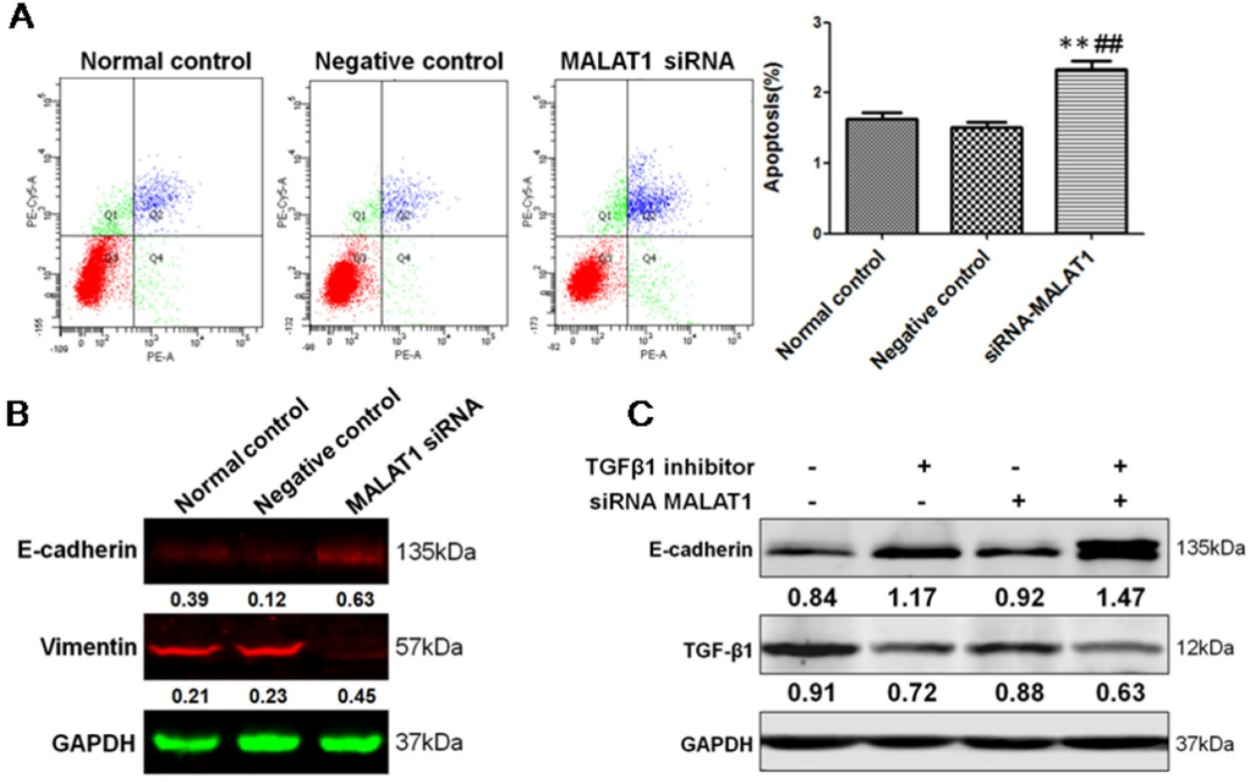
** MALAT1 promoted invasion of ESCC via TGF-β1-induced EMT.** (A) FCM showed that knockdown of MALAT1 significantly enhanced cell apoptosis. (B) Expression of E-cadherin and Vimentin after MALAT1 knockdown were detected by Western blot. (C) Western blot analysis of the expression of E-cadherin after knockdown of MALAT1 and TGF-β1 inhibition. (**: compared with normal control,* P*<0.01; ##: compared with negative control, *P*<0.01).

To define the functional links between MALAT1 and EMT, we first examined the effects of MALAT1 knockdown on E-cadherin and Vimentin expression. As shown in Fig. 6 and Fig. 7, MALAT1 knockdown significantly enhanced E-cadherin expression and reduced Vimentin expression at the protein level in Eca109 cells. More importantly, knockdown of MALAT1 significantly suppressed cell proliferation and migration, reduced invasion, and enhanced cell apoptosis (Fig. 6, Fig. 7, Fig. s1, Fig. s2). These results demonstrated that MALAT1 promoted invasion of ESCC via EMT. As we have already shown, TGF-β1 can induce EMT in ESCC, and MALAT1 is an important promoter of EMT; however, direct evidence that MALAT1 promotes TGF-β1-induced EMT in ESCC is still lacking. Here, MALAT1 siRNA was transfected into ESCC cells after TGF-β1 inhibitor treatment. Although TGF-β1 inhibition increased the expression of E-cadherin, knockdown of MALAT1 significantly increased the expression of E-cadherin after TGF-β1 inhibition (Fig. 7C). These results confirmed our hypothesis that MALAT1 promotes TGF-β1-induced EMT.

## Discussion

ESCC is considered one of the leading causes of cancer-related deaths in China, due to its high incidence and aggressive nature. Although surgery and chemoradiotherapy are widely used in ESCC patients, their prospects for full recovery are still poor. Due to the lack of effective therapeutic options that could enhance the patient survival rate, an increasing number of studies are focused on the mechanism of invasion and metastasis in ESCC. In varying ways, EMT induced by TGF-β plays an important role in the progression of invasion and metastasis of ESCC. However, the pathological significance and the underlying process of EMT still need to be investigated. Akt is likely to have an important role in EMT induced by TGF-β1 in ESCC and may contribute to invasion and metastasis 28. Li and his group reported that ESCC tissues exhibited upregulated expression of TGF-β1, especially in Kazakh patients in Xinjiang, China 28, 29. TGF-β stimulated the smad2/3 signaling pathway, inducing EMT and consequently leading to malignant changes. In this study, we analyzed Kazakh's ESCC patients with high expression of TGF-β1 who have a poor prognosis. Furthermore, our results indicated that lncRNA MALAT1 participates in the progression of TGF-β1-induced EMT.

Upregulation of MALAT1 has been found in various cancers and more recently in ESCC. The up-regulation of MALAT1 in ESCC tissues can impact the degree of tumor progression and is predictive of postoperative survival 30. Although its dysregulation is considered a marker for metastatic development, the functional role of MALAT1 in this process is only beginning to emerge. The mechanism by which MALAT1 contributes to cancer progression is through induction of EMT. Fan Y and colleagues showed that upregulation of MALAT1 promotes bladder cancer metastasis induced by TGF-β 31. Wang Y et al 32 evaluated expression variation of MALAT1 after treated with TGF-β, finding that TGF-β significantly induced malat1 levels in human head and neck squamous cell carcinoma cells; subsequent similar work by Du MY et al 33 detected MALAT1 expression variation in 6-10B cells treated with TGF-β by way of qRT-PCR, showing that TGF-β treatment increased MALAT1 expression in nasopharyngeal carcinoma. The two independent studies fundamentally established the relationship between TGF-β1 and MALAT1 in outline; although the detailed regulation remains to be fleshed out with more data and evidence. To investigate the role of MALAT1 in Endothelial-to-mesenchymal transition (EndMT) of human circulating endothelial progenitor cells (EPCs) induced by TGF-β1, Xiang Y et al 34 treated cells with TGF-β1 and found that TGF-β1 strongly induced MALAT1 mRNA expression in EPCs. However, the regulated correlation between TGF-β and MALAT1 in ESCC has not been revealed. Based on these research findings, we postulated that TGF-β may regulate the expression of MALAT1, thereby promoting invasion of ESCC induced by EMT. Here, we demonstrated that TGF-β1 induces MALAT1 expression and EMT in ESCC cells. The expression of MALAT1 is significantly correlated with the expression of TGF-β1 35. Moreover, MALAT1 inhibition increases E-cadherin expression with concurrent downregulation of Vimentin in ESCC cells. Furthermore, MALAT1 inhibition decreases cell proliferation, migration and invasion and increases cell apoptosis in ESCC. More importantly, MALAT1 inhibition partly abrogates TGF-β1-induced EMT.

Another mechanism by which MALAT1 positively regulates metastasis is through the regulatory effects of various factors 36, 37. Recent studies showed that MALAT1 promotes malignant development of ESCC by targeting β-catenin via Ezh2 35, as well as by modifying the ATM-CHK2 pathway 38. Moreover, posttranscriptional regulation of MALAT1 by miR-101 and miR-217 exists in ESCC cells 39, 40. Although we did not investigate the downstream target gene of MALAT1 in ESCC, we preliminarily predicted that 113 miRNAs could be regulated by MALAT1 (supplementary table 1), including miR-26a 41, 42 and miR-106b 43, 44, which we have already demonstrated are significantly correlated with invasion and metastasis of ESCC. Thus, MALAT1 may regulate EMT in ESCC through the regulatory effects of downstream miRNAs.

In conclusion, we replicated the overexpression of TGF-β1 in ESCC, which indicates poor prognosis. MALAT1 may promote ESCC invasion and metastasis by targeting TGF-β1-induced EMT, although other mechanisms could not be excluded.

## Supplementary Material

Supplementary figures and tables.

## Figures and Tables

**Table 1:** Expression of EMT markers in Kazakh's ESCC and normal adjacent tissue (NAT)

Item	Type	Positive (%)	Negative (%)	*P*
TGF-β1	ESCC	62 (86.0)	10 (14.0)	0.000*
	NAT	29 (39.0)	43 (61.0)	
E-cadherin	ESCC	29 (40.3)	43 (59.7)	0.000*
	NAT	52 (72.2)	20 (27.8)	
Vimentin	ESCC	40 (55.6)	32 (44.4)	0.007*
	NAT	27 (37.5)	45 (62.5)	

*P < 0.05.

**Table 2:** Correlation between E-cadherin and pathological parameters

Pathological parameters Item	N	Positive (%)	Negative (%)	*P*
**Differentiation**				0.018*
High	40	20 (50.0)	20 (50.0)	
Moderate	26	9 (34.6)	17 (65.4)	
Low	6	0 (0)	6 (100.0)	
**Lymphatic metastasis**				0.972
No	25	10 (40.0)	15 (60.0)	
Yes	47	19 (40.4)	28 (59.6)	
**Depth of infiltration**				0.704
Mucosa and muscular layer	23	10 (43.5)	13 (56.5)	
Serous layer	49	19 (38.8)	30 (61.2)	

*P < 0.05.

## Abbreviations

EC: esophageal cancer
ESCC: esophageal squamous cell carcinoma
TGF-β1: transforming growth factor β1
EMT: epithelial-mesenchymal transition
LncRNA: long noncoding RNA
MALAT1: metastasis-associated lung adenocarcinoma transcript 1
FFPE: formalin-fixed paraffin-embedded
NAT: normal adjacent tissue
SPSS: statistical product and service solutions
