# TGF-β1-Induced Upregulation of MALAT1 Promotes Kazakh's Esophageal Squamous Cell Carcinoma Invasion by EMT: Erratum

**DOI:** 10.7150/jca.73596

**Published:** 2022-04-25

**Authors:** Qing Liu, Shutao Zheng, Yumei Chen, Tao Liu, Xiujuan Han, Xiao Zhang, Tongxue Shen, Xiaomei Lu

**Affiliations:** 1 Clinical Medical Research Institute, First Affiliated Hospital of Xinjiang Medical University, Xinjiang Uygur Autonomous Region, Urumqi, PR China.; 2 State Key Laboratory of Pathogenesis, Prevention, Treatment of High Incidence Diseases in Central Asian, Xinjiang Uygur Autonomous Region, Urumqi, PR China.; 3 Health Management Center, Xinjiang Medical University, Xinjiang Uygur Autonomous Region, Urumqi, PR China.

In our paper 1, we come to realize that Figure 4D was mistakenly taken for Figure 3C that was the same as Figure 4D when preparing the manuscript. The corrected Figure 4D was provided below. The correction made in this erratum will not affect the results and conclusions we drew. We apologize for this error and for any inconvenience that may bring about to the readers and the editors of the journal.

## Figures and Tables

**Figure 4:**
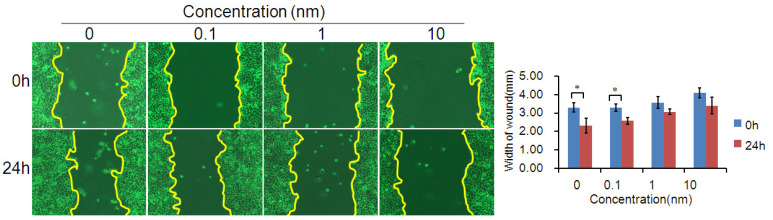
(D) The migratory variations of ESCC cells were analyzed using a wound healing assay after treatment with TGF-β1 inhibitor. Magnification: 100× (*, P<0.05).

## References

[1] Liu Q, Zheng S, Chen Y, Liu T, Han X, Zhang X, Shen T, Lu X (2020). TGF-β1-Induced Upregulation of MALAT1 Promotes Kazakh's Esophageal Squamous Cell Carcinoma Invasion by EMT. J Cancer.

