# Supplementary material for: TGF-β1-Induced Upregulation of MALAT1 Promotes Kazakh's Esophageal Squamous Cell Carcinoma Invasion by EMT
Source: J Cancer. 2020 Oct 4;11(23):6892–901. doi: 10.7150/jca.48426 (PMC7592017; doi:10.7150/jca.48426)
Supplement: Supplementary file 1 — Supplementary figures and tables. [file jcav11p6892s1.pdf]

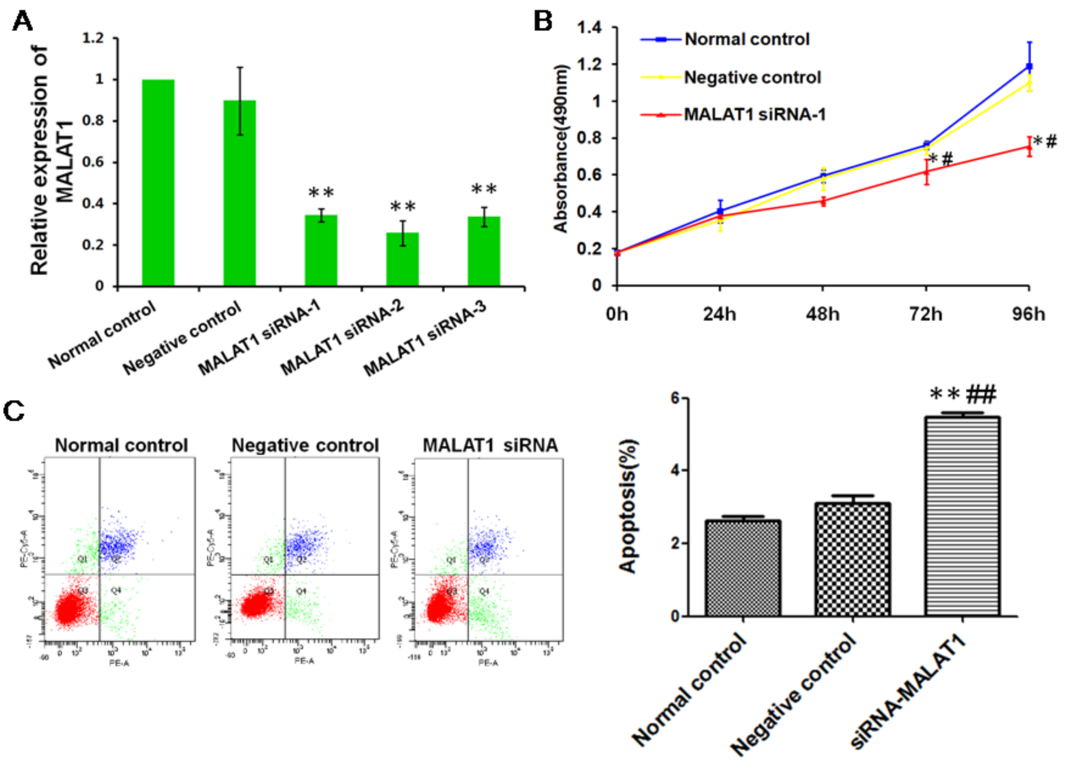

Supplementary Figure 1. MALAT1 promoted proliferation of ESCC in another ESCC cell line. (A) qRT-PCR showed that MALAT1 siRNA significantly reduced expression of MALAT1 in KYSE150 cell; (B) MTT assays revealed that MALAT1 knockdown significantly reduced the growth rate of KYSE150 cell; (C) FCM showed that knockdown of MALAT1 significantly enhanced cell apoptosis. (**: compaired with normal control, $P < 0.01$; ##: compaired with negative control, $P < 0.01$).

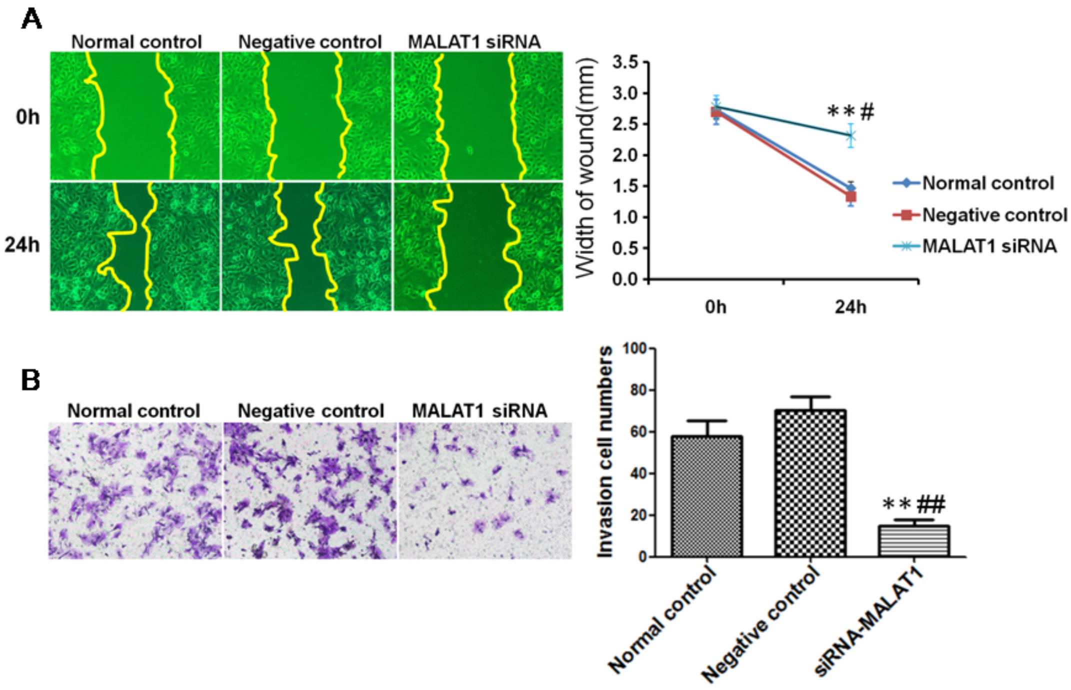

Supplementary Figure 2. MALAT1 promoted invasion of ESCC in another ESCC cell line. (A) migratory variation of KYSE150 was analyzed using wound healing assay after MALAT1 knockdown. Magnification: 100×; (B) Transwell assay showed the cell invasive variation of KYSE150 after MALAT1 knockdown.Magnification:200×. (**: compaired with normal control, $P < 0.01$; ##: compaired with negative control, $P < 0.01$).

Supplementary Table1. Predicted miRNAs regulated by MALAT1.

name	mirAccession	geneName
hsa-miR-200b-3p	MIMAT0000318	MALAT1
hsa-miR-200a-3p	MIMAT0000682	MALAT1
hsa-miR-429	MIMAT0001536	MALAT1
hsa-miR-378f	MIMAT0018932	MALAT1
hsa-miR-30e-5p	MIMAT0000692	MALAT1
hsa-miR-30c-5p	MIMAT0000244	MALAT1
hsa-miR-101-3p	MIMAT0000099	MALAT1
hsa-miR-197-3p	MIMAT0000227	MALAT1
hsa-miR-320b	MIMAT0005792	MALAT1
hsa-miR-92b-3p	MIMAT0003218	MALAT1
hsa-miR-181b-5p	MIMAT0000257	MALAT1
hsa-miR-181a-5p	MIMAT0000256	MALAT1
hsa-miR-135b-5p	MIMAT0000758	MALAT1
hsa-miR-205-5p	MIMAT0000266	MALAT1
hsa-miR-194-5p	MIMAT0000460	MALAT1
hsa-miR-346	MIMAT0000773	MALAT1
hsa-miR-146b-5p	MIMAT0002809	MALAT1
hsa-miR-378c	MIMAT0016847	MALAT1
hsa-miR-202-3p	MIMAT0002811	MALAT1
hsa-miR-129-5p	MIMAT0000242	MALAT1
hsa-miR-708-5p	MIMAT0004926	MALAT1
hsa-miR-3167	MIMAT0015042	MALAT1
hsa-miR-200c-3p	MIMAT0000617	MALAT1
hsa-miR-141-3p	MIMAT0000432	MALAT1
hsa-miR-613	MIMAT0003281	MALAT1
hsa-miR-26a-5p	MIMAT0000082	MALAT1
hsa-miR-135a-5p	MIMAT0000428	MALAT1
hsa-miR-320d	MIMAT0006764	MALAT1
hsa-miR-1297	MIMAT0005886	MALAT1
hsa-miR-17-5p	MIMAT0000070	MALAT1
hsa-miR-20a-5p	MIMAT0000075	MALAT1
hsa-miR-92a-3p	MIMAT0000092	MALAT1
hsa-miR-4306	MIMAT0016858	MALAT1
hsa-miR-370-3p	MIMAT0000722	MALAT1
hsa-miR-494-3p	MIMAT0002816	MALAT1
hsa-miR-376a-3p	MIMAT0000729	MALAT1
hsa-miR-376b-3p	MIMAT0002172	MALAT1
hsa-miR-544a	MIMAT0003164	MALAT1
hsa-miR-485-5p	MIMAT0002175	MALAT1
hsa-miR-154-5p	MIMAT0000452	MALAT1
hsa-miR-203a	MIMAT0000264	MALAT1
hsa-miR-211-5p	MIMAT0000268	MALAT1

hsa-miR-422a	MIMAT0001339	MALAT1
hsa-miR-328-3p	MIMAT0000752	MALAT1
hsa-miR-140-5p	MIMAT0000431	MALAT1
hsa-miR-22-3p	MIMAT0000077	MALAT1
hsa-miR-144-3p	MIMAT0000436	MALAT1
hsa-miR-142-3p	MIMAT0000434	MALAT1
hsa-miR-338-3p	MIMAT0000763	MALAT1
hsa-miR-320c	MIMAT0005793	MALAT1
hsa-miR-1	MIMAT0000416	MALAT1
hsa-miR-23a-3p	MIMAT0000078	MALAT1
hsa-miR-181c-5p	MIMAT0000258	MALAT1
hsa-miR-181d-5p	MIMAT0002821	MALAT1
hsa-miR-150-5p	MIMAT0000451	MALAT1
hsa-miR-125a-3p	MIMAT0004602	MALAT1
hsa-miR-519d-3p	MIMAT0002853	MALAT1
hsa-miR-4429	MIMAT0018944	MALAT1
hsa-miR-4262	MIMAT0016894	MALAT1
hsa-miR-217	MIMAT0000274	MALAT1
hsa-miR-216a-5p	MIMAT0000273	MALAT1
hsa-miR-216b-5p	MIMAT0004959	MALAT1
hsa-miR-26b-5p	MIMAT0000083	MALAT1
hsa-miR-149-5p	MIMAT0000450	MALAT1
hsa-miR-499a-5p	MIMAT0002870	MALAT1
hsa-miR-124-3p	MIMAT0000422	MALAT1
hsa-miR-155-5p	MIMAT0000646	MALAT1
hsa-miR-185-5p	MIMAT0000455	MALAT1
hsa-miR-378i	MIMAT0019074	MALAT1
hsa-miR-378b	MIMAT0014999	MALAT1
hsa-miR-425-5p	MIMAT0003393	MALAT1
hsa-miR-28-5p	MIMAT0000085	MALAT1
hsa-miR-378d	MIMAT0018926	MALAT1
hsa-miR-367-3p	MIMAT0000719	MALAT1
hsa-miR-3139	MIMAT0015007	MALAT1
hsa-miR-143-3p	MIMAT0000435	MALAT1
hsa-miR-145-5p	MIMAT0000437	MALAT1
hsa-miR-378a-3p	MIMAT0000732	MALAT1
hsa-miR-378h	MIMAT0018984	MALAT1
hsa-miR-146a-5p	MIMAT0000449	MALAT1
hsa-miR-378e	MIMAT0018927	MALAT1
hsa-miR-1271-5p	MIMAT0005796	MALAT1
hsa-miR-206	MIMAT0000462	MALAT1
hsa-miR-30a-5p	MIMAT0000087	MALAT1
hsa-miR-4465	MIMAT0018992	MALAT1
hsa-miR-4644	MIMAT0019704	MALAT1

hsa-miR-590-3p	MIMAT0004801	MALAT1
hsa-miR-25-3p	MIMAT0000081	MALAT1
hsa-miR-93-5p	MIMAT0000093	MALAT1
hsa-miR-106b-5p	MIMAT0000680	MALAT1
hsa-miR-96-5p	MIMAT0000095	MALAT1
hsa-miR-383-5p	MIMAT0000738	MALAT1
hsa-miR-320a	MIMAT0000510	MALAT1
hsa-miR-30b-5p	MIMAT0000420	MALAT1
hsa-miR-30d-5p	MIMAT0000245	MALAT1
hsa-miR-491-5p	MIMAT0002807	MALAT1
hsa-miR-876-5p	MIMAT0004924	MALAT1
hsa-miR-873-5p	MIMAT0004953	MALAT1
hsa-miR-204-5p	MIMAT0000265	MALAT1
hsa-miR-23b-3p	MIMAT0000418	MALAT1
hsa-miR-32-5p	MIMAT0000090	MALAT1
hsa-miR-455-5p	MIMAT0003150	MALAT1
hsa-miR-4770	MIMAT0019924	MALAT1
hsa-miR-23c	MIMAT0018000	MALAT1
hsa-miR-374b-5p	MIMAT0004955	MALAT1
hsa-miR-374a-5p	MIMAT0000727	MALAT1
hsa-miR-384	MIMAT0001075	MALAT1
hsa-miR-363-3p	MIMAT0000707	MALAT1
hsa-miR-20b-5p	MIMAT0001413	MALAT1
hsa-miR-106a-5p	MIMAT0000103	MALAT1
hsa-miR-503-5p	MIMAT0002874	MALAT1
hsa-miR-506-3p	MIMAT0002878	MALAT1
hsa-miR-224-5p	MIMAT0000281	MALAT1
